# Modeling Functional Limitations, Gait Impairments, and Muscle Pathology in Alzheimer’s Disease: Studies in the 3xTg-AD Mice

**DOI:** 10.3390/biomedicines9101365

**Published:** 2021-10-01

**Authors:** Lidia Castillo-Mariqueo, M. José Pérez-García, Lydia Giménez-Llort

**Affiliations:** 1 Institut de Neurociències, Universitat Autònoma de Barcelona, 08193 Barcelona, Spain; lidia.castillom@e-campus.uab.cat; 2 Department of Psychiatry and Forensic Medicine, School of Medicine, Universitat Autònoma de Barcelona, 08193 Barcelona, Spain; 3 Department of Neuroscience, Vall d’Hebron Research Institute, 08035 Barcelona, Spain; maria.perez@vhir.org

**Keywords:** translational neuroscience, Alzheimer’s disease, gait, muscular strength, muscular endurance, motor performance, frailty

## Abstract

Gait impairments in Alzheimer’s disease (AD) result from structural and functional deficiencies that generate limitations in the performance of activities and restrictions in individual’s biopsychosocial participation. In a translational way, we have used the conceptual framework proposed by the International Classification of Disability and Health Functioning (ICF) to classify and describe the functioning and disability on gait and exploratory activity in the 3xTg-AD animal model. We developed a behavioral observation method that allows us to differentiate qualitative parameters of psychomotor performance in animals’ gait, similar to the behavioral patterns observed in humans. The functional psychomotor evaluation allows measuring various dimensions of gait and exploratory activity at different stages of disease progression in dichotomy with aging. We included male 3xTg-AD mice and their non-transgenic counterpart (NTg) of 6, 12, and 16 months of age (n = 45). Here, we present the preliminary results. The 3xTg-AD mice show more significant functional impairment in gait and exploratory activity quantitative variables. The presence of movement limitations and muscle weakness mark the functional decline related to the disease severity stages that intensify with increasing age. Motor performance in 3xTg-AD is accompanied by a series of bizarre behaviors that interfere with the trajectory, which allows us to infer poor neurological control. Additionally, signs of physical frailty accompany the functional deterioration of these animals. The use of the ICF as a conceptual framework allows the functional status to be described, facilitating its interpretation and application in the rehabilitation of people with AD.

## 1. Introduction

Alzheimer’s disease (AD) is a complex and heterogeneous disorder with a distinctive clinical presentation [1,2]. Motor and sensory alterations are less frequent but can appear in intermediate and advanced stages of the disease [3]. Although the main signs of AD are cognitive impairment, motor disorders such as bradykinesia, rigidity, and gait disorders are of great importance due to the functional limitations and impairments that they cause during the disease [4,5]. In this sense, different studies have demonstrated different motor alterations during the last two decades, particularly those associated with walking and displacement [6,7,8]. Thus, gait disorders in AD patients have been described within the group of alterations known as “frontal gait” and, in particular, gait in AD has been defined as “cautious gait” [5,9,10]. This gait pattern is similar to that observed in the aging; there may be decrease in speed, stride length, and postural stability of gait, which is manifested more specifically in static and dynamic balance, with a widened base of support [11].

Walking (gait) constitutes a biological activity of the human being [12]. It is complex, learned, and begins with a voluntary act [13]. The mode of locomotion allows one to move in a vertical position without getting too tired; it is composed of three essential phases: support, double support, and swing [14,15]. It implies a dynamic balance, which is constantly lost and recovered each time a step is performed [9,14]. While the bodyweight is supported by one leg, the other swings forward to initiate the next support, this action being fluid, rhythmic, and automatically synchronized [16]. Achieving these steps makes it possible to reveal different patterns that can determine the healthy state of gait related to the physical health of individuals and, in particular, of older adults [17,18].

The gait pattern evolves through the different age groups; thus, in adolescents and young adults, it is characterized by a certain lightness, flexibility, and agility, qualities that will diminish as the years go by [19,20,21]. During aging, these parameters will change around 60 and 70 years of age, where the physiological process causes these changes to be progressive and of varying severity depending on the degree of alterations that may occur [20]. Even healthy people over the age of 65 show some decrease in performance on the timed walking test (TUG) and the 6 min walk test (6MWT) [22]. Lower mobility test scores in healthy older people have been shown to predict the development of future mobility limitations [23].

On the other hand, muscle strength is relevant to gait performance [24]. Some studies have reported that older adults’ decreased muscle strength and gait speed were associated with poor cognition [25,26]. From the early stages of AD, a decrease in muscle strength can be observed even without loss of muscle mass, progressing to a loss of both in moderate stages [27]. However, optimal muscle strength and physical activity level are related to better performance on cognitive and learning tests in older adults with mild to moderate cognitive impairment who live in nursing homes [28]. 

In this way, sarcopenia is closely related to dementia, particularly AD, although few studies examine its prevalence and associated factors [27]. Poor muscle function, but not reduced lean muscle mass, drives the association of sarcopenia with cognitive decline in old age [29]. It needs more scientific studies that identify the characteristics of the muscular structure related to sarcopenia that identifies older adults at risk of cognitive deterioration in old age.

The present work proposes translating the International Classification of Functioning, Disability, and Health (ICF) conceptual framework to classify and describe functioning and disability in gait and exploratory activity in the 3xTg-AD animal model and its non-transgenic counterpart with normal aging. We developed a method for the characterization of qualitative parameters of psychomotor performance in the gait pattern of male mice in different stages of the disease: initial (6 months), advanced beta (12 months), and advanced beta-tau (16 months) in contrast with normal aging.

## 2. Materials and Methods

### 2.1. Animals

A total of forty-five homozygous 3xTg-AD (*n* = 24) and non-transgenic (NTg, *n* = 21) male mice of 6, 12, and 16 months of age in a C57BL/6J background (the transfer is accomplished by at least ten cycles of backcrossing) established at the Universitat Autònoma de Barcelona [30] were used in this study. As previously described, the 3xTg-AD mice harboring transgenes were genetically modified at the University of California at Irvine [31]. Animals were kept in groups of 3–4 mice per cage (Macrolon, 35 × 35 × 25 cm, Panlab, SL, Barcelona, Spain) filled with 5 cm of clean wood cuttings (Ecopure, Chips6, Date Sand, UK; uniform cross-sectional wood granules with 2.8–1.0 mm chip size) and nesting materials (Kleenex, Art: 08834060, 21 cm × 20 cm, White). In all cases, standard home cages covered with a metal grid allow the perception of olfactory and auditory stimuli from the rest of the colony. All animals were kept under standard laboratory conditions of food and water ad libitum, 20 ± 2 °C, 12 h light cycle: dark with lights on at 8:00 a.m. and 50–60% relative humidity. All procedures followed the Spanish legislation on “Protection of animals used for experimental and other scientific purposes” and the EU Directive (2010/63/EU) on this issue. The study complies with the ARRIVE guidelines developed by the NC3Rs and aims to reduce the number of animals used [32].

### 2.2. Experimental Design

A cross-sectional study of 3 cohorts of 3xTg-AD and NTg male mice was carried out. The first group includes 6-month-old mice of both genotypes, the second 12-month-old, and the third 16-month-old. They were temporarily included in two batches once they reached the required study age. The evaluation of the gait and exploratory activity was carried out in two evaluation days.

### 2.3. Behavioral Assessments

**Figure 1:**
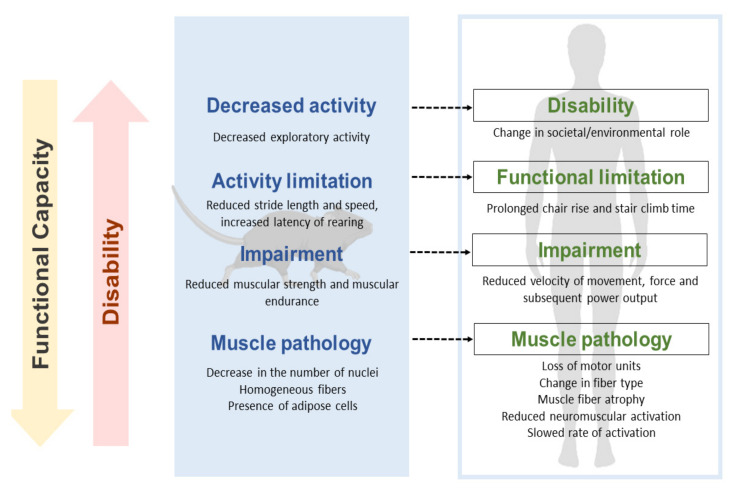
Proposal for a translational approach to motor dysfunction at different levels of disability. The figure details in a translational way the equivalence of the disability process from the 3xTg-AD mouse model to that expected in humans. It exemplifies a pathway that links pathology, deficiencies, functional limitations, and disability of gait and exploration (it has been adapted from Verbrugge and Jette, 1994; and Reid and Fielding, 2012) [34,35].

In a translational approach, the concepts proposed in ICF have been incorporated to describe the factors that functionally intervene in spontaneous gait and exploratory activity. They have been included in this way: “Activity” describes the shape and quantification of the trajectory and displacement. “Body function” allows a description of the movement pattern and the associated muscular strength. Finally, ”Body structure” gives an account of the state in which muscle groups and joints are found in our object of study. In the same way, we have included the concepts that account for disability in the assessed tasks, activity limitations, and body function and structure impairments (see Figure 1). 

Behavioral evaluations were performed in two days. During the morning, the tests were carried out; 30 min were allowed to habituate the animals in the test room before starting the measurements. The evaluation protocol, bizarre behaviors registered, the physical phenotype of frailty, gait, and Rotarod used here were recently reported in Castillo-Mariqueo and Gimenez-Llort’s 2021 study [33]. In addition, videos of gait were taken for posterior analysis with KINOVEA version 0.8.15 free software.

#### 2.3.1. Activity—Spontaneous Gait and Exploration

The animals’ spontaneous gait and exploratory activity were filmed from the undersurface [36] and a transverse plane that allowed the registration of the legs and the trajectory during the test. Each mouse was placed in a 27.5 × 9.5 cm transparent housing box, and gait performance was directly observed for one minute. The KINOVEA 8.15 software was used for the quantification and analysis of the gait trajectory.

Quantitative parameters of gait. The quantitative parameters were measured according to the Castillo-Mariqueo and Gimenez-Llort protocol 2021 [33]. Stride length, stride length variability, speed, and acceleration were included according to the methodology used by Wang et al. in 2017 [35].

Exploration includes body position, limb support, and moving around. The exploratory activity was recorded in parallel with spontaneous walking through video analysis and direct observation. For one minute, freezing or movement latency, the latency of the first rearing, the number of scans on the horizontal axis (corners visited), and scans on a vertical axis were recorded, taking the hind legs (rearings) as reference. During the tests, defecation and urination were also recorded.

#### 2.3.2. Body Function—Mobility and Muscular Strength

General mobility includes bizarre gait patterns and freezing. For the discrimination of bizarre gait patterns, the trajectory of each route was differentiated according to the form of displacement, being able to be straight, scanning, backward, or circling. Then, the postural pattern of movement is differentiated: normal, shrink, or stretching. The movements were visually analyzed through videos and direct observation in the trajectory of the forward displacement in the transverse and sagittal planes. Their presence or absence was recorded for each one [33].

Forelimb grip strength-Hanger Test. The muscular strength of the forelimbs was measured using the hanger test, which is based on the tendency of a mouse to grasp a bar when suspended by the tail instinctively. We have replicated the methodology previously described by our group [33,37]. Muscle strength was measured on the second day of assessment.

#### 2.3.3. Body Structure—Joints and Muscles

Joints to detect kyphosis. Kyphosis was differentiated into postural and structural, according to the analysis method established in previous research by our laboratory [38]. Kyphosis was measured during spontaneous locomotion and later confirmed in postural inspection (using joints and thoracic–lumbar structure as references), assigning a score from 0 to 2, where 0 indicates the absence of kyphosis, 1 indicates postural kyphosis, and 2 indicates structural kyphosis.

Muscles related to sarcopenia. The animals were sacrificed and subjected to necropsy to extract the quadriceps and triceps surae muscles, and these were subsequently weighed individually. The “sarcopenia index” [38] was applied to obtain an indirect measure of sarcopenia as a biological marker of frailty.

Morphological features of quadriceps and triceps surae. A qualitative evaluation of longitudinal sections of quadriceps and triceps surae stained with hematoxylin and eosin (H&E) was performed. The muscles were dissected and fixed with 10% formalin (Sigma-Aldrich, Saint Louis, MO, USA) for 24 h and then embedded in paraffin for further analysis. Histological sections of 5 μm were stained with a standard H&E method. We used the standard protocol of the Laboratory of Microscopic Anatomy of the Department of Morphological Sciences of the Autonomous University of Barcelona. Sections were first re-hydrated by passing them through decreasing concentrations of Ethanol (EtOH) (absolute and 96°). The sections were incubated in Harris hematoxylin (Merck-Sigma Aldrich, St. Louis, MO, USA) for 10 min and washed under running water for 5 min. Because Mayer Hematoxylin was used, a de-differentiation process was needed, with consecutively Et-OH-HCl (2.5%) and ammonia water (0.3%) solutions. After this, sections were incubated in Eosin Y (Merck-Sigma Aldrich) for 5 min, previously acidified with glacial acetic acid. Finally, the sections were dehydrated by passing them through increasing concentrations of EtOH and cleared in Xylol, and mounted with DPX (PanReac AppliChem, ITW reagents, IL, USA). The images were taken with 20X objective on a Nikon Eclipse 80i microscope, using a digital camera running on the control software ACT-1 (ver2.70) (Nikon Instruments Inc., Tokyo, JP). Characteristics of location and number of nuclei, fibers’ distribution and shape, and adipose tissue were identified. A generic description and qualifier of intensity were given with five levels represented by (+) for the quantity graduation.

#### 2.3.4. Motor Performance, Geotaxis, and Hindlimb Clasping

Additionally, motor learning, physical endurance, geotaxis, and hindlimb clasping were evaluated to show functional impairment related to gait and exploratory activity, according to the protocol developed by Castillo-Mariqueo, Giménez-Llort, 2021 [33]. In the present work, we have adopted the protocol combining learning and physical endurance. Thus, motor learning was evaluated in the constant mode and physical resistance in the accelerated mode of the Rotarod apparatus (Ugo basile^®^, Mouse RotaRod NG). We recorded the number of trials until reaching over 60 s of permanence on the wheel to measure learning. Subsequently, after 2 min of rest, we carried out a single trial in the accelerated mode to assess physical endurance.

### 2.4. Statistics

Statistical analyses were performed using SPSS 15.0 software. Results were expressed as the mean ± standard error of the mean (SEM) for each task and trial. Gait, exploration, forelimb grip strength, muscles (sarcopenia), motor performance, and geotaxis were analyzed with one-way ANOVA followed by post hoc Bonferroni. In addition, the effect of Genotype (G) and Age (A) in each of them was identified. The incidence and prevalence of body position, general mobility, kyphosis, and hindlimb clasping were analyzed using the Chi-square test or Fisher’s exact test. Additionally, the relationship between activity limitation and restriction (presence/absence) with stride length, speed, and cadence was analyzed with the Point-Biserial Correlation. The horizontal and vertical exploration and rearing latency were also related to the deficiencies in exploration and gait. The survival curve of both genotypes was analyzed with the Kaplan–Meier test (Lon Rank). In all cases, statistical significance was considered at *p* < 0.05.

## 3. Results

As shown in Table 1, we have proposed a translational approach for the interpretation of the results obtained in the measurement of gait and exploratory activity in the 3xTg-AD mouse model in different stages of the disease and its counterpart NTg of normal aging, according to the analysis and quantification parameters proposed by ICF. 

### 3.1. Activity—Spontaneous Gait and Exploration 

**Figure 2:**
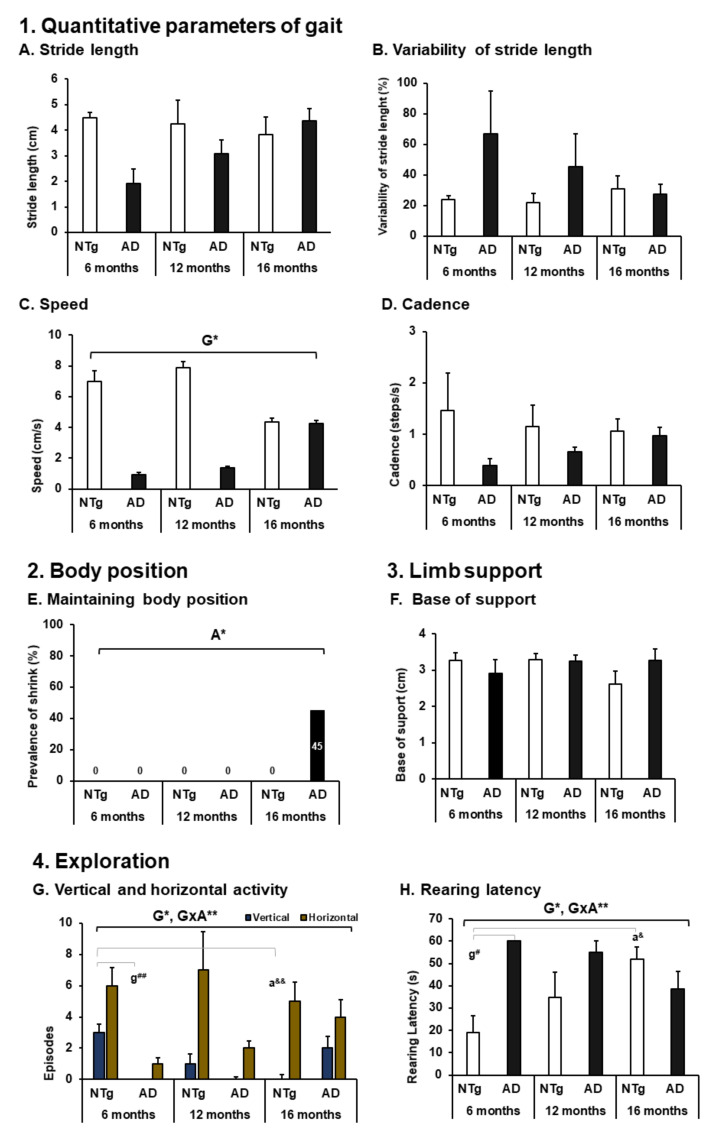
ACTIVITY: Spontaneous gait and exploration. 1. Quantitative parameters of gait; (**A**) stride length; (**B**) variability of stride length; (**C**) speed; (**D**) cadence. 3. Limb support. (**F**) Base of support. 4. Exploration. (**G**) Vertical and horizontal activity; (**H**) rearing latency. The results are expressed as mean ± SEM. Statistics: one-way ANOVA, Age effect expressed as (**A**); Genotype effect expressed as (**G**); Genotype and Age interaction effect is expressed as (GxA); * *p* < 0.05, ** *p* < 0.01 followed by post hoc Bonferroni test, * *p* < 0.05, ** *p* < 0.01,; differences between NTg vs. 3xTg-AD are expressed (g): ^#^ *p* < 0.05, ^##^ *p* < 0.01; differences between age in each group are expressed (a): ^&^ *p* < 0.05, ^&&^ *p* < 0.01. 2. Body position; (**E**) maintaining body position, the results are expressed as prevalence (%). Statistics: Fisher’s exact test, Age effect are expressed as (**A**), Genotype effect are expressed as (**G**); Genotype and Age interaction effect are expressed as (GxA); * *p* < 0.05 and ** *p* < 0.01.

As illustrated in Figure 2, the quantitative parameters of gait show a tendency to increase stride length in 3xTg-AD animals, although they are not statistically significant (stride length (cm), NTg 6 months: 4.48 ± 0.20; NTg 12 months: 4.24 ± 0.95; NTg 16 months: 3.84 ± 0.68; 3xTg-AD 6 months: 1.92 ± 0.57; 3xTg-AD 12 months: 3.09 ± 0.54; 3xTg-AD 16 months: 4.36 ± 0.48). Although the differences between NTg ages do not reach statistical significance, we can observe that stride length remains relatively stable as age increases, while in the 3xTg-AD group they present differences between the ages since the 95% confidence interval does not overlap between 6 months, 12 months, and 16 months (6 months, 1.92 + 0.57 = 2, 49; 12 months, 3.09 − 0.57 = 2.55; 16 months, 3.84 + 0.68 = 4.52).

On the other hand, in the exploration, the animals differed in their performance in horizontal and vertical activity (horizontal activity, ANOVA F (5,39) = 2.427, *p* = 0.050; vertical activity, ANOVA F (5,39) = 4.600, *p* = 0.002). Genotype differences can be noted in each age group with a lower performance in 3xTg-AD animals (horizontal activity: genotype differences, ANOVA F (1.44) = 9.548, *p* = 0.004). Likewise, we can evidence a genotype difference in vertical activity (vertical activity: genotype differences, ANOVA F (1,44) = 7.209, *p* = 0.011) and a significant difference at the age of 6 months between the groups (Bonferroni post hoc: NTg 6 months vs. 3xTg-AD 6 months, *p* = 0.004). It was also detected that at an older age in the normal aging group there is a decrease in vertical activity (Bonferroni post hoc: NTg 6 months vs. NTg 16 months, *p* = 0.012). In turn, the genotype per age interaction (GxA) show the decrease in the vertical exploratory activity of NTg versus 3xTg-AD (GxA, ANOVA, F (2.43) = 8.519, *p* = 0.001), see Figure 2G (Exploration). The first time they perform vertical activity (latency of the first rearing) is also determined by the genotype and its interaction with age, highlighting that the group of 3xTg-AD mice at the age of 6 months did not register this activity and that in the animals NTg latency increases with age progressively (rearing latency, ANOVA F (5,39) = 4120, *p* = 0.004 post hoc NTg 6 months vs. 3xTg-AD 6 months, *p* = 0.010; NTg 6 months vs. NTg 16 months, *p* = 0.026. Genotype differences, ANOVA F (1.44) = 6.443, *p* = 0.015, GxA differences, F (2.43) = 8330 *p* = 0.001) (see Figure 2H (Eploration)).

### 3.2. Body Function—Mobility and Muscular Strength

**Figure 3:**
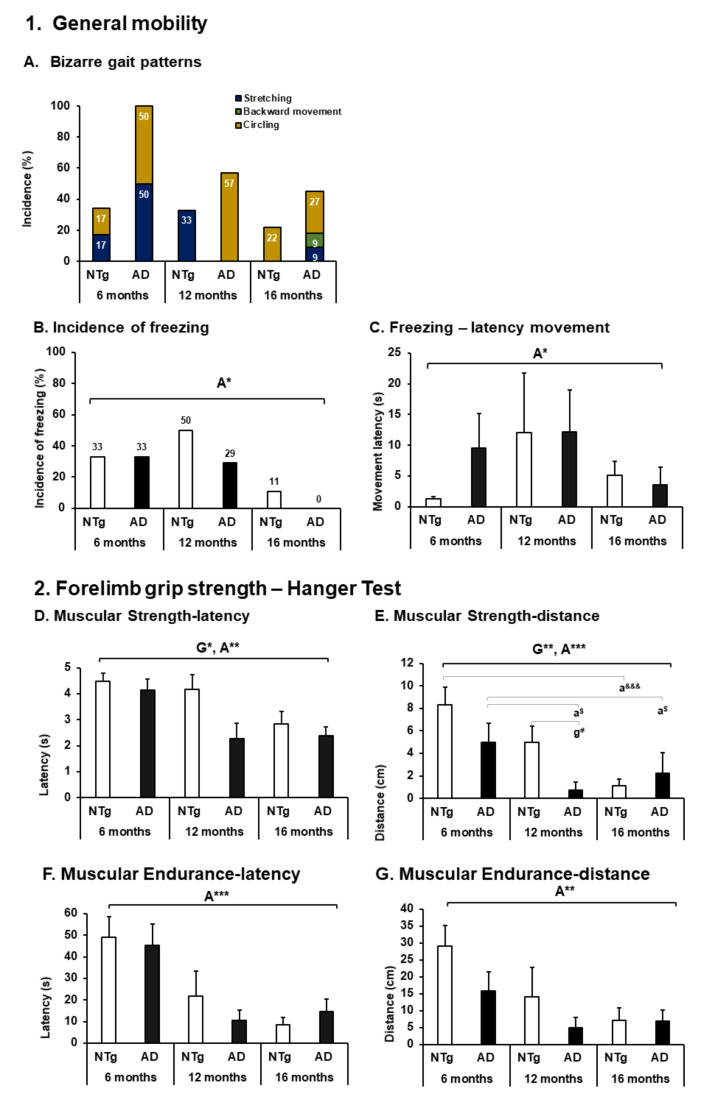
BODY FUNCTION—mobility and forelimb grip strength. 1. General mobility. (**A**) Bizarre gait patterns; (**B**) incidence of freezing; the results are expressed as incidence (%). Statistics: Fisher’s exact test, Age effect is expressed as (**A**); Genotype effect is expressed as (**G**); Genotype and Age interaction effect is expressed as (GxA); * *p* < 0.05, ** *p* < 0.01 and *** *p* < 0.001. (**C**) Freezing—latency movement; 2. Forelimb grip strength—Hanger Test. (**D**) Muscular Strength—latency; (**E**) Muscular Strength—distance; (**F**) Muscular Endurance—latency; (**G**) Muscular Endurance—distance, the results are expressed as mean ± SEM. Statistics: one-way ANOVA, Age effect expressed as (**A**); Genotype effect expressed as (**G**); Genotype and Age interaction effect is expressed as (GxA). * *p* < 0.05, ** *p* < 0.01, and *** *p* < 0.001 followed by post hoc Bonferroni test, * *p* < 0.05, ** *p* < 0.01, and *** *p* < 0.001; differences between NTg vs. 3xTg-AD are expressed (g): ^#^ *p* < 0.05; differences between age in NTg group are expressed (a): ^&&&^ *p* < 0.001, and for 3xTg-AD group are expressed (a): ^$^ *p* < 0.01.

As shown in Figure 3, the animals exhibited a series of bizarre behaviors called bizarre gait patterns. There is a high incidence of circling in 3xTg-AD animals (3xTg-AD: 6 months 3/6 (50%), 12 months 4/7 (57%), 16 months 3/11 (27%)); despite not being statistically significant, its presence can modify performance in gait and exploration, which are described later (see Figure 5). It can also be seen that this behavior appears in NTg animals with a lower incidence (NTg: 6 months 1/6 (17%), 16 months 2/9 (22%)). Stretching (NTg: 6 months 1/6 (17%), 12 months 2/6 (33%)—3xTg-AD: 6 months 3/6 (50%), 16 months 1/11 (9%)], and in animals 3xTg-AD 16 months backward movement [1/11 (17%)), see Figure 3A. In addition, a high incidence of freezing was evidenced in which as age increases, its incidence decreases regardless of genotype (Fisher exact test, *p* = 0.032). It is also appreciated that the time invested in this behavior varies with age, being less at 16 months without genotype effect and higher at 12 months (ANOVA, F (2.43) = 3.473 *p* = 0.041), (see Figure 3B,C).

**Figure 4:**
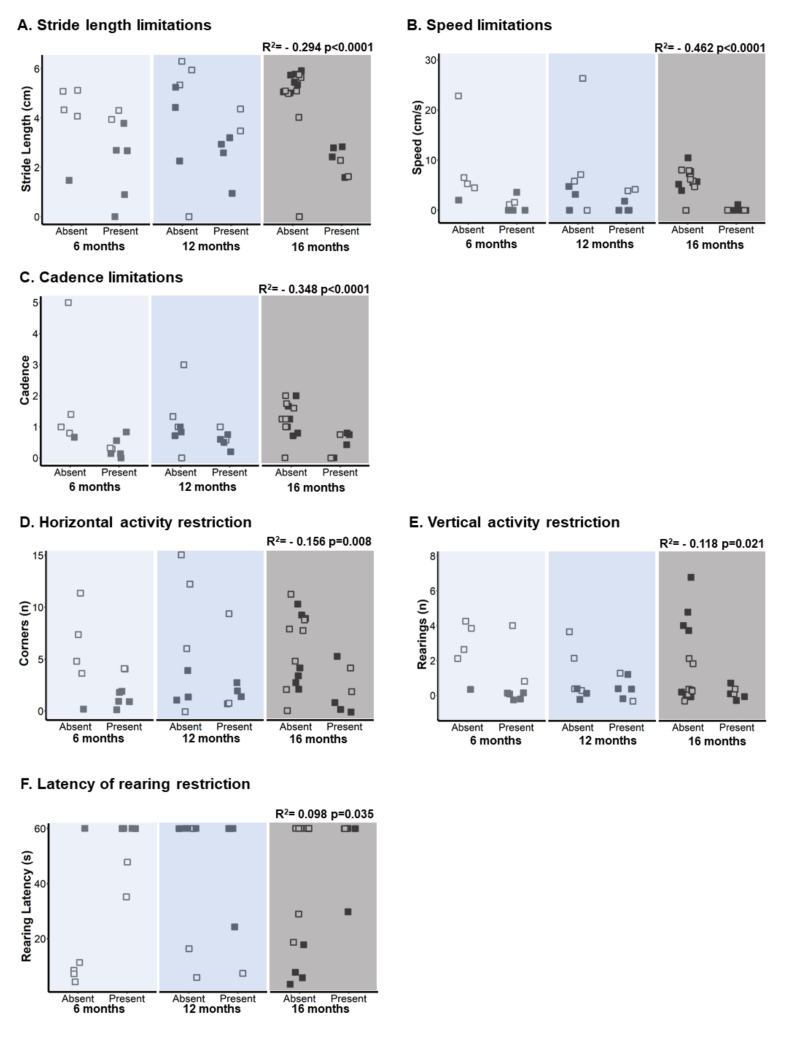
Activity limitations and restrictions of gait and exploration. (**A**) Stride length limitations, (**B**) speed limitations, (**C**) cadence limitations, (**D**) horizontal activity restriction, (**E**) vertical activity restriction, and (**F**) latency of rearing restriction. The NTg group has been represented by a white square and the 3xTg-AD group by a black square. According to the groups under study, it has been defined as “present/absent” the behaviors reported as bizarre gait patterns of each animal. The Point-Biserial Correlation has been applied to determine the relationship between the activity limitation and restriction (presence/absence) with stride length, speed, and cadence and exploration. Statistics: Pearson r2.

Additionally, we have detected a correlation between the incidence of these behaviors and performance in gait and exploration, as shown in Figure 4. Thus, the variables stride length, speed, and cadence negatively correlate with the presence of bizarre gait pattern, causing limitation in the displacement and trajectory of gait (stride length, Pearson: r2 = (−) 0.294 *p* < 0.0001. Speed, Pearson: r2 = (−) 0.462 *p* < 0.0001. Cadence, Pearson: r2 = (−) 0.348 *p* < 0.0001), see Figure 4A–C. While the horizontal and vertical exploration variables correlate negatively, rearing latency does so positively with the presence of bizarre gait pattern, which leads to a restriction to the performance of these behaviors (horizontal activity, Pearson r2 = (−) 0.156 *p* = 0.008. Vertical activity, Pearson: r2 = (−) 0.118 *p* = 0.021. Rearing latency, Pearson: r2 = 0.098 *p* = 0.035) (see Figure 4D–F).

Muscle strength, on the other hand, was lower in older animals, and the transgenic genotype at each age was lower than the non-transgenic genotype at all ages (Muscular strength—latency: ANOVA F (5.39) = 4.385, *p* = 0.003; Age effect, F (2, 43) = 5.702, *p* = 0.007; Genotype effect, F (1,44) = 5.895, *p* = 0.020). In the same way, it can be noted that the distance reached when the animals move on the bar was less as age increases and the 3xTg-AD mice were lower than the NTg at 6 and 12 months; otherwise, it occurs at 16 months, but it is not statistically significant (muscular strength—distance: ANOVA F (5.39) = 9.847, *p* < 0.0001 post hoc NTg 6 months vs. NTg 16 months *p* < 0.0001; 3xTg-AD 6 months vs. 3xTg-AD 16 months *p* = 0.023; 3xTg-AD 6 months vs 3xTg-AD 12 months *p* = 0.050; NTg 12 months vs 3xTg-AD 12 months, *p* = 0.045). Age effect F (2.43) = 17,320 *p* < 0.0001. Genotype effect F (1.44) = 11.786 *p* = 0.001], see Figure 3D,E. At the same time, the muscular endurance and the distance of displacement was determined by the age of the animals decreasing as the age increases in both groups (muscular endurance—latency: ANOVA F (5,39) = 3.296, *p* = 0.014. Age effect, F (2,43) = 8,154, *p* = 0.001. Muscular endurance—distance ANOVA F (5.39) = 3.394, *p* = 0.012. Age effect, F (2.43) = 7.295, *p* = 0.002) (see Figure 3F,G).

### 3.3. Body Structure—Joints and Muscles

**Figure 5:**
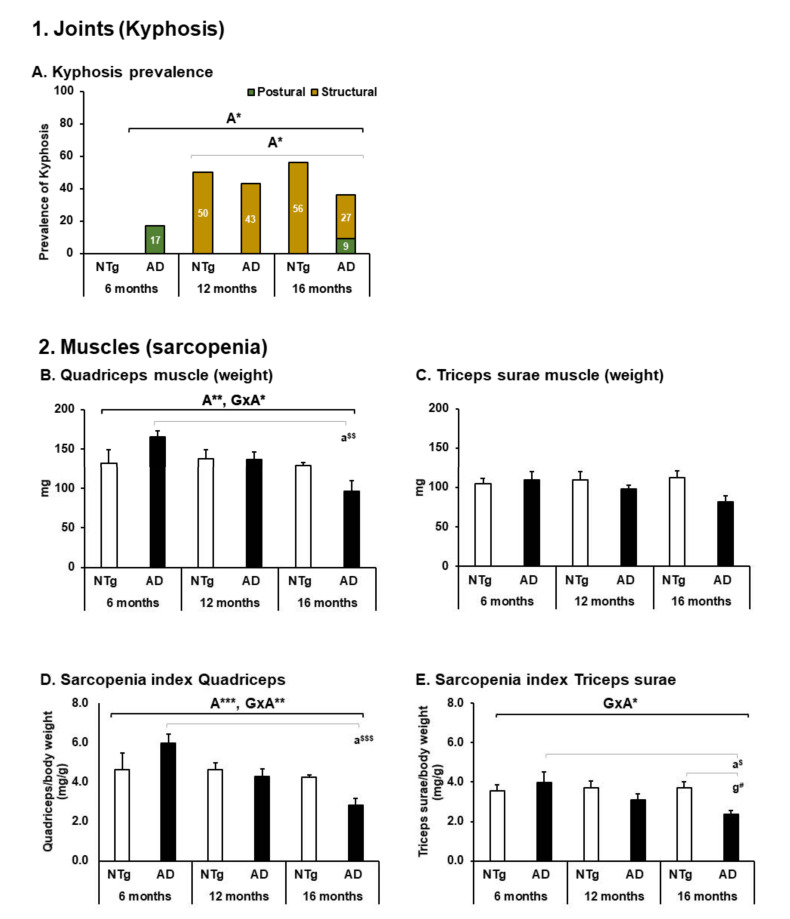
BODY STRUCTURE—joints and muscles. 1. Joints (Kyphosis), (**A**) kyphosis prevalence, the results are expressed as prevalence (%). Statistics: Fisher’s exact test; Age effect is expressed as (**A**); * *p* < 0.05. 2. Muscles (sarcopenia), (**B**) quadriceps muscle (weight); (**C**) triceps surae muscle (weight); (**D**) Sarcopenia index Quadriceps; (**E**) Sarcopenia index Triceps surae; the results are expressed as mean ± SEM. Statistics: one-way ANOVA, Age effect expressed as (**A**); Genotype effect expressed as (**G**); Genotype and Age interaction effect is expressed as (GxA). * *p* < 0.05, ** *p* < 0.01 and *** *p* < 0.001 followed by post-hoc Bonferroni test, * *p* < 0.05, ** *p* < 0.01, and *** *p* < 0.001; differences between NTg vs. 3xTg-AD are expressed (g): ^#^ *p* < 0.05, differences between age in 3xTg-AD group are expressed (a): ^$^ *p* < 0.01, ^$$^ *p* < 0.01, and ^$$$^ *p* < 0.001.

The most prevalent postural alteration was kyphosis, with structural kyphosis having the highest incidence in older animals regardless of genotype (Kyphosis prevalence, age differences Fisher exact test *p* = 0.025. Structural kyphosis incidence, age differences *p* = 0.016), see Figure 5A. This joint deformation was observed at the thoracolumbar level.

**Figure 6:**
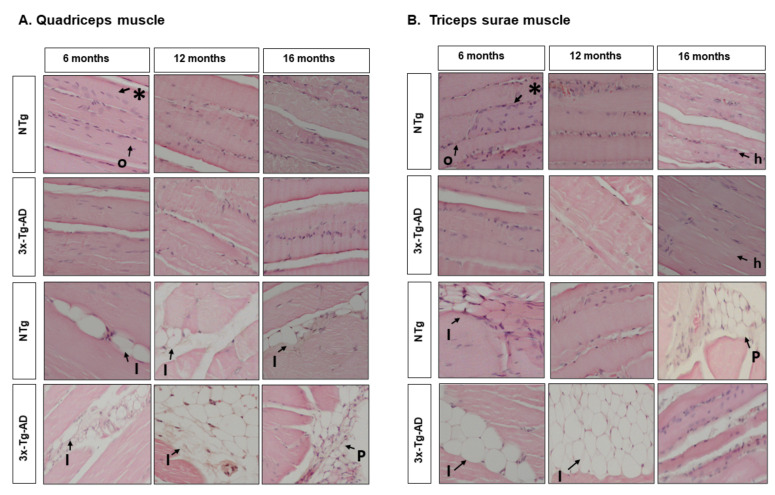
Morphological comparison of muscle tissue in normal and AD-pathological aging. Hematoxylin and eosin-stained horizontal sections of muscles. (**A**) Quadriceps muscle; (**B**) Triceps surae muscle. Representative H&E images of longitudinal skeletal muscle at 6, 12, and 16 months. Symbols indicate the morphological features, as follows: *—Peripheral nuclei; o—homogeneous fibre distribution; h—heterogeneous fibre distribution; I—intramuscular adipose tissue; P—peripheral adipose tissue. The images were taken with 20× objective lens; the scale bar represents 0.32 µm.

At the level of muscle tissue, the quadriceps presented variations in weight, with a tendency to decrease with age determined by the GxA interaction and a significant decrease between the 3xTg-AD of 6 months versus 16 months (quadriceps, ANOVA F (5, 39) = 4.314, *p* = 0.003, post hoc 3xTg-AD 6 months vs. 3xTg-AD 16 months, *p* = 0.001. Age effect, F (2.43) = 5.715, *p* = 0.007. GxA effect, F (2.43) = 4.291, *p* = 0.021), see Figure 5B. In the triceps surae muscle, no statistically significant differences were detected; it can be seen that all groups, regardless of age, seem to maintain a similar weight range, see Figure 5C. When applying the indirect measure of sarcopenia, the differences in quadriceps were maintained (sarcopenia Index—quadriceps: ANOVA F (5.39) = 6.705, *p* < 0.0001, post hoc 3xTg-AD 6 months vs. 3xTg-AD 16 months *p* < 0.0001. Age effect F (2.43) = 9.693, *p* < 0.0001. GxA effect F (2.43) = 5.623, *p* = 0.007), see Figure 5D. On the other hand, when applying this method in the triceps sural muscle, it was possible to distinguish a GxA interaction effect, where at six months, the 3xTg-AD mice present greater weight and decrease with age, and in the case of the NTg, this is maintained stable (sarcopenia index—triceps surae: ANOVA F (5.39) = 4.160 *p* = 0.004 post hoc 3xTg-AD 6 months vs. 3xTg-AD 16 months *p* = 0.010. NTg 16 months vs. 3xTg-AD 16 months *p* = 0.020. GxA effect, F (2.43) = 3.917 *p* = 0.028), see Figure 5E. Figure 6A,B illustrates the morphological characteristics of the quadriceps and triceps surae. Table 2 depicts the characteristics of the nucleus, fiber, and adipose tissue.

### 3.4. Motor Performance, Geotaxis, and Hindlimb Clasping

**Figure 7:**
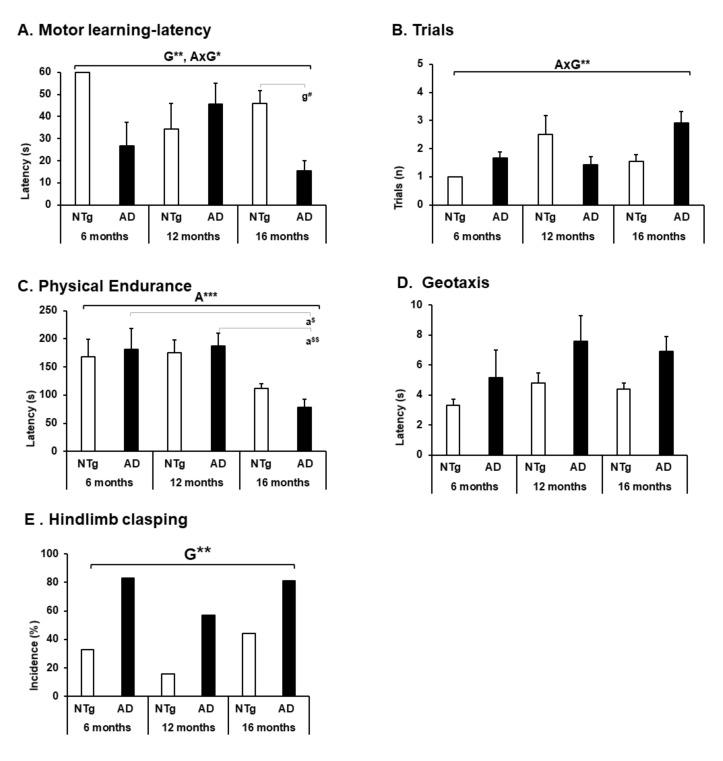
Motor performance, geotaxis and hindlimb clasping. (**A**) Motor learning—latency; (**B**) trials; (**C**) physical endurance; (**D**) geotaxis; the results are expressed as mean ± SEM. Statistics: one-way ANOVA, Age effect expressed as (**A**); Genotype effect expressed as (G); Genotype and Age interaction effect are expressed as (GxA). * *p* < 0.05, ** *p* < 0.01, and *** *p* < 0.001 followed by post hoc Bonferroni test, ^#^ *p* < 0.05; differences between age in 3xTg-AD group are expressed (a): ^$^ *p* < 0.01, ^$$^ *p* < 0.01.(**E**) Hindlimb clasping; the results are expressed as prevalence (%). Statistics: Fisher’s exact test; Age effect is expressed as (A); Genotype effect is expressed as (G); Genotype,* *p* < 0.05.

Motor performance and physical performance were evaluated in other tests to obtain a complete analysis regarding the psychomotor abilities of the animals. Thus, motor learning showed an interaction between the genotype factor and GxA, highlighting a low performance in 3xTg-AD mice at the age of 6 and 16 months, concerning NTg of the same age (motor learning—latency, ANOVA F (5.39) = 4.995, *p* = 0.001 post hoc NTg 16 months vs. 3xTg-AD 16 months *p* = 0.026. Genotype effect F (1.44) = 7.926, *p* = 0.008. GxA effect F (2, 43) = 5.184, *p* = 0.010. Trials, ANOVA F (5.39) = 3.953, *p* = 0.005. GxA F (2.43) = 5.454 *p* = 0.008) (see Figure 7A,B). On the other hand, physical endurance decreases with age, with 16 months being the age with the lowest performance in both groups, but statistically significant in 3xTg-AD mice (physical endurance, ANOVA F (5.39) = 5.189, *p* = 0.001 post hoc 3xTg-AD 6 months vs. 3xTg-AD 16 months, *p* = 0.017; 3xTg-AD 12 months vs. 3xTg-AD 16 months, *p* = 0.006. Age effect F (2.43) = 11.371, *p* < 0.0001) (see Figure 7C). Geotaxis did not show statistical differences, but a higher latency was observed in the 3xTg-AD animals in each age group (see Figure 7D). In the hindlimb clasping test, we can highlight a significant genotype difference in each age group with a higher incidence of this sign in each 3xTg-AD mice (hindlimb clasping, Fisher exact test genotype = 0.007). 

### 3.5. Survival, Kyphosis, and Frailty Phenotype

Table 3 shows the survival, kyphosis, and frailty phenotype of the mice at each age. For the survival analysis, we carried out a follow-up from birth to 16-month-old of the siblings of the sample included in the study, completing a cohort of 115 male mice. Logarithmic rank analysis shows a significant genotype-dependent difference (χ2 (1) = 8.045, *p* = 0.005) with a higher mortality rate in NTg mice in each age group (6-month-old: NTg 3/15 (20%); 3xTg-AD 0/15. 12-month-old: NTg 3/9 (33.3%), 3xTg-AD 1/16 (6.2%). 16-month-old: NTg 20/40 (50%); 3xTg-AD 5/24 (20.8%)). On the other hand, kyphosis presents a higher incidence as age increases without genotype differences (Kyphosis (absent/present) Fisher exact test (5) = 10.694, *p* = 0.052. Age, Fisher exact test (2) = 10.070, *p* = 0.007. Genotype n.s). While postural kyphosis does not show significant differences between the groups, structural kyphosis increases its prevalence at ages 12 and 16 months of age independent of genotype (Fisher’s exact test (2) = 8.464, *p* = 0.016). In the same way, body weight increases with age in the case of 3xTg-AD mice and is maintained in the case of NTg, with 3xTg-AD mice presenting greater weight at 16-month-old compared to NTg 16-month-old (Age effect, ANOVA F (2.44) = 3.268, *p* = 0.049; 3xTg-AD 12-month-old vs 16-month-old, *p* = 0.037). Regarding the physical conditions that the animals presented, no differences were detected in alopecia. On the other hand, body position, palpebral closure, and tail position were characteristics only present in the older group of 3xTg-AD mice (body position, Fisher’s exact test (5) = 10.036, *p* = 0.006. Age effect, Fisher’s exact test (2), *p* = 0.046. Palpebral closure, Fisher’s exact test (5) = 7.493, *p* = 0.037. Tail position, Fisher’s exact test (5) = 7.493, *p* = 0.037). Piloerection was present in NTg mice at the age of 12 and 16 months in contrast to 3xTg-AD mice, where its presentation appears at 16 months (Fisher (5) = 10.047, *p* = 0.027. Age effect, Fisher’s exact test (2) = 8.338, *p* = 0.010).

Finally, tremor shows differences in genotype and age, presenting a high incidence at 16 months in 16-month-old mice (tremor, Fisher’s exact test (5) = 23.346, *p* < 0.0001. Age effect, Fisher’s exact test (2) = 10.170, *p* = 0.005. Genotype effect, χ2 (1) = 6.945, *p* = 0.012).

## 4. Discussion

In contrast to the huge literature on the AD-associated hallmark impairment in cognitive domains, gait disorders in Alzheimer’s disease are an emerging field. They result from structural and functional deficiencies that generate limitations in the performance of activities and also imply restrictions in the biopsychosocial participation of individuals [39,40,41,42,43]. Evidence suggests that AD has a long preclinical phase, during which its characteristic pathology accumulates, and the patient’s function diminishes considerably [42,44]. Motor problems have been described as occurring early in the AD process, rather than being a feature exclusively related to end-stage AD pathology [45,46]. 

At the translational level, in animal models, we have recently described alterations in the trajectory and displacement that interfere with gait and exploratory activity have recently been reported in middle-aged (13-month-old) and old (16-month-old) male C57BL/6 and 3xTg-AD mice, which in the mutant corresponds to ages mimicking advanced and very advanced stages of the disease [33]. Furthermore, these alterations increase their incidence in endpoint situations at different ages regardless of the studied genotype [38,47,48]. In this report, we have expanded the study of functionality and disability described for humans to provide a translational proposal, which allows us to differentiate dysfunctions, gait disorders, and exploration in the 3xTg-AD model at different stages of disease progression and as compared to C57BL/6 with normal aging. As shown in Figure 1, the functional limitations that we have detected are equivalent to the difficulties that an older adult typically faces when carrying out their activities of daily living and that we can consider as markers of functional health deterioration.

### 4.1. Activity—Spontaneous Gait and Exploration 

Particularly in gait, the variable speed, as in humans [43,49], seems to be the variable with the highest sensitivity to detect impairments of displacement and locomotion. Furthermore, when bizarre gait patterns (circling, backward movement, stretching) are present, stride length, speed, and cadence decrease in performance regardless of age and genotype. On the other hand, 6-month-old 3xTg-AD mice have a shorter stride length that increases with age. This result may be related to the novelty situation, where we have detected a higher incidence of freezing (no movement) and higher episodes of bizarre gait patterns in this group. Furthermore, it has previously been reported that in 3xTg-AD mice aged from 10 to 14 months, the stride length is greater than that of control mice [49]. The authors point out that a possible explanation for this difference is the differences between species, where quadruped locomotion seems to have compensatory mechanisms that intervene even after injury at the brain level [50], mediating that the kinematic parameters can be preserved. In contrast, it can be inferred that older animals present a favorable indicator in their gait performance, and this may be related to survival and individual characteristics.

A study conducted in 3- and 24-month-old male C57BL/6 mice found that aged mice exhibited significantly lower cadence and decreased stride time variability [50]. They also reported that aging tended to alter footstep patterns, for which they associated with aging the alterations that occur in gait [50]. There are also technological devices and software to make possible the equivalence of some human signatures in mice, highlighting those related to gait disorders in Parkinson’s disease [41,51,52,53]. However, studying whole body gait and posture in rodent models requires specialized methods and remains a challenge if other motivational or emotional response behavioral factors are integrated, which is the case of some Alzheimer’s disease models where a noticeable neuropsychiatric-like pattern is exhibited. In this sense, in the face of novelty situations, 3xTg-AD mice respond with neophobia and anxiety-like behaviors [54,55], whereas in humans, they have been reported from initials stages of the disease [56]. Neophobia modifies the exploratory activity as age increases, accentuating the symptoms [57]. However, we have described that there’s a relationship between bizarre gait patterns and horizontal and vertical components of exploratory activity. Thus, bizarre gait patterns limiting locomotion in 3xTg-AD mice do the same in NTg mice, which tends to increase with age. In 3xTg-AD mice, these behaviors are mainly related to psychiatric and neurological disorders [30,37,58]. However, bizarre behaviors can be heterogeneous and have a low incidence in males compared to females, as described by Baeta-Corral and Giménez-Llort, 2014 [30]. Therefore, in this sex, these behaviors emerge at early stages and progress with the disease similar to that observed in the bizarre swimming patterns in the Morris water maze, where we have described the presence of circling appears at early stages (6 months of age) [59] and worsens with age [60,61]. 

### 4.2. Body Function—Mobility and Muscular Strength

General mobility was interfered with by periods of freezing. We can distinguish that the 12-month-old animals presented several freezing episodes in both genotypes. At the age of 6 months, the group of 3xTg-AD mice presented a long freezing behavior, taking longer to perform the first movement, which can also influence the decrease in exploration and the quantitative parameters of the gait, similar to what happens in scenarios of social isolation [33].

At the level of muscle strength, in humans, it has been described that the decrease in strength in the initial stages of AD does not imply changes at the muscle fiber level [61,62,63,64]. However, in intermediate stages, it could be accompanied by a decrease in the number of muscle fibers that in advanced stages are reflected in sarcopenia associated with loss of muscle strength [35,63]. Our results showed that the decrease in muscle strength would be associated with aging, as occurs with muscular endurance. Nevertheless, at the age of 12 months, there is a drop in grip strength in 3xTg-AD mice. It has also been reported that at six months, 3xTg-AD mice have a deficit in grip strength [65], but at 16 months, these results are not reproduced [66]. In isolation, the 3xTg-AD mice show a conserved strength at the age of 13 months over the mice that lived in groups [33]. These findings may point to the heterogeneity of aging and the stage of AD in which muscle strength is measured.

### 4.3. Body Structure—Joints and Muscles

At the same time, postural patterns such as shrinkage and structural changes at the joint level of the thoracolumbar spine accompany 3xTg-AD mice with a high incidence of structural kyphosis [33,38,48]. Our results show that both 3xTg-AD and C57BL/6 mice show an increase in the incidence of structural kyphosis after 12 months of age, which could explain, from a postural point of view, the decrease in exploration in both groups as age increases.

As the weight of the quadriceps muscle shows, there is a progressive decrease in 3xTg-AD mice that, unlike the C57BL/6 controls in which it appears to be attenuated, a higher weight range is preserved even in older animals. In contrast, both groups maintain a similar weight of triceps muscle at 12 and 16 months of age. A study in C57BL/6J females reported a progressive weight loss from 15 months in the quadriceps muscle, which is considerably accentuated at 24 months [66]. Similarly, a decrease in muscle weight over 25 months has been reported in male C57BL/6J mice in the gastrocnemius and soleus muscle [67].

Furthermore, we have applied an indirect measure of sarcopenia to verify its presence to investigate these findings further. In the quadriceps muscle, aging is related to sarcopenia, while in the transgenic group, sarcopenia appears at 16 months. Interestingly, the triceps surae muscle also indicates sarcopenia in 16-month-old 3xTg-AD mice. Using this measure, a study conducted in female C57BL/6J mice concluded that sarcopenia would be present at around 24 months in the quadriceps muscle [68]. However, in male C57BL/6J mice, it could occur at earlier ages, reporting 20 months as the age of most significant change in the gastrocnemius muscle [69]. 

In natural aging models of the C57BL/6J strain, it has been reported that the primary phenotype of sarcopenia is a decrease in muscle mass and a decrease in the cross-sectional area of muscle fibers [70,71,72]. The optimal age of study would be 25 months [67,72]. Thus, we found the fibers are distributed homogeneously, with differences between them, but maintain a similar distribution. However, a difference is observed in the number of nuclei in NTg control animals that seems higher than in 3xTg-AD, especially at 12 months. We also found the presence of adipose cells, which exhibited a different distribution for each muscle type. Thus, adipose cells were present to a lesser extent in quadriceps, independently of genotype and age, with an intramuscular predominance. Oppositely, adipose cells exhibited a peripheral or intramuscular localization according to the genotype and age in the triceps. Thus, in the NTg control group, adipose cells were found in more peripheral areas, with a more significant proportion at 16 months.

In contrast, in the 3xTg-AD group, the adipose cells were more intramuscular, and a higher proportion was found at 12 months. Interestingly, the NTg control mice had a similar weight at each age, while the weight of 3xTg-AD mice increased with age.

### 4.4. Motor Performance, Geotaxis, and Hindlimb Clasping

On the other hand, we have measured the animals’ motor learning and physical resistance to obtain a global vision regarding their psychomotor performance. We have shown that in the advanced stage of the disease, 3xTg-AD mice have a lower-than-expected performance that is replicated in motor learning and physical endurance. Similarly, in C57BL/6 control animals, the observed changes are more attenuated due to aging. In the case of geotaxis, an increase in turning latency was found in the 3xTg-AD group, which, although it did not present significant differences, could indicate a poor use of postural and balance strategies to regain the verticality of their body on the grille in which they are located. For its part, hindlimb clasping showed a higher incidence in the 3xTg-AD group without being associated with the stages of disease progression. This particular sign can indicate the severity of the motor impairment that the mice present [73,74,75].

### 4.5. Survival, Kyphosis, and Frailty Phenotype

Finally, we can point out that C57BL/6 control animals have a higher mortality ratio in all age groups regarding survival, consistent with previous studies [76]. As the frailty phenotype shows, some signs of deterioration are related to one group or another. In the case of 3xTg-AD mice, physical and postural conditions appear to be the highest incidence, and in their NTg counterparts, piloerection and tremor, which in both groups, were found similarly increased with age. These variables indicate the general state of the mice without interfering with their functional performance of the gait and exploration that we have reported.

## 5. Conclusions

According to the literature, this is the first report that comprehensively presents the gait disturbances and functional limitations in the exploratory activity of the 3xTg-AD mouse model and, as compared to C57BL/6 with normal aging, uses a conceptual model that allows translation to humans. The use of the ICF as a conceptual framework allows describing the functional state, facilitating its interpretation and application in the rehabilitation of people with AD.

In summary, the main conclusions are:

(1) The 3xTg-AD mice show more significant functional impairment in gait and exploratory activity quantitative variables.
(2) The presence of movement limitations and muscle weakness mark the functional decline related to the disease severity stages that intensify with increasing age.
(3) Motor performance in 3xTg-AD is accompanied by a series of bizarre behaviors that interfere with the trajectory, which allows us to infer poor neurological control.
(4) Signs of physical frailty accompany the functional deterioration of these animals.
(5) Signs of sarcopenia are present in an advanced stage of AD, with differences in fibre distribution, number of cell nuclei, and presence of adipose tissue.

## Figures and Tables

**Table 1:** Proposal to functional analysis to mimics capacities and disabilities from humans in mice.

Translational Functioning and Disability (ICF)						
**ACTIVITY—Spontaneous gait and exploration**	**NTg Mice**			**3xTg-AD Mice**		
**1. Quantitative parameters of gait (see Figure 2)**	**6 Months**	**12 Months**	**16 Months**	**6 Months**	**12 Months**	**16 Months**
A. Stride length (cm)	ABSENT	MILD	MILD	MODERATE	MODERATE	MILD
B. Variability of stride length (%)	ABSENT	ABSENT	MILD	SEVERE	MODERATE	MODERATE
C. Speed (cm/s)	ABSENT	ABSENT	MILD	SEVERE	SEVERE	MODERATE
D. Cadence (steps/s)	ABSENT	ABSENT	ABSENT	SEVERE	MODERATE	MODERATE
**2. Body position (see Figure 2)**						
E. Maintaining body position (%*)*	ABSENT	ABSENT	ABSENT	ABSENT	ABSENT	MILD
**3. Limb support**						
F. Base of support (cm)	ABSENT	ABSENT	MILD	ABSENT	ABSENT	ABSENT
**4. Exploration (see Figure 2)**						
G. Vertical and horizontal activity (n episodes)	MILD	MILD	MILD	MODERATE	SEVERE	MILD
H. Rearing latency (s)	MILD	MODERATE	SEVERE	COMPLETE	SEVERE	MODERATE
**BODY FUNCTION—mobility and forelimb grip strength**						
**1. General mobility (see Figure 3)**						
A. Bizarre gait patterns (incidence %)	MILD	MODERATE	MILD	MODERATE	MODERATE	MILD
B. Freezing (movement latency)	ABSENT	MODERATE	MILD	MODERATE	MODERATE	MILD
C. Freezing—latency movement	ABSENT	MODERATE	MILD	MODERATE	MODERATE	MILD
**2. Forelimb grip strength—Hanger Test (see Figure 3)**						
D. Muscular Strength (latency)	ABSENT	MILD	MODERATE	MILD	MODERATE	MODERATE
E. Muscular Strength (distance)	ABSENT	MILD	SEVERE	MILD	SEVERE	MODERATE
F. Muscular Endurance (latency)	ABSENT	MODERATE	SEVERE	MILD	SEVERE	SEVERE
G. Muscular Endurance (distance)	ABSENT	MODERATE	SEVERE	MODERATE	SEVERE	SEVERE
**BODY STRUCTURE—joints and muscles**						
**1. Joints (see Figure 4)**						
A. Kyphosis prevalence	ABSENT	MODERATE	MODERATE	MILD	MILD	MILD
**2. Muscles (see Figure 4)**						
B. Quadriceps muscle (weight)	MILD	MILD	MILD	ABSENT	MILD	MODERATE
C. Triceps surae muscle (weight)	MILD	MILD	MILD	MILD	MILD	MODERATE
D. Sarcopenia index—Quadriceps	MILD	MILD	MILD	ABSENT	MILD	MODERATE
E. Sarcopenia index—Triceps	MILD	MILD	MILD	MILD	MILD	MODERATE
***Qualifiers:*** Generic qualifier with the negative scale used to indicate the extent or magnitude of an impairment: NO impairment, (absent) 0–4%; MILD impairment, (slight, low) 5–24%; MODERATE impairment, (medium, fair) 25–49%; SEVERE impairment, (high, extreme), 50–95%; COMPLETE impairment, (total) 96–100%—not specified in the ICF for humans. Activity limitations are difficulties an individual may have in executing activities: NO difficulty, (absent) 0–4%; MILD difficulty, (slight, low) 5–24%; MODERATE difficulty, (medium, fair) 25–49%; SEVERE difficulty, (high, extreme) 50–95%; COMPLETE difficulty, (total) 96–100%—not specified in the ICF for humans.						

Table 1. Outcome measures that link gait and exploration impairments and limitations of male 3xTg-AD mice at different stages of AD progression to describe the functioning, according to qualifiers of ICF. Translational Functioning and Disability: ACTIVITY—spontaneous gait and exploration, BODY FUNCTION—mobility and forelimb grip strength, and BODY STRUCTURE—joints and muscles. Absent (green), Mild (light green), Moderate (yellow), Severe (orange), and Complete (red).

**Table 2:** Morphological features of quadriceps and triceps surae.

Morphological Features	NTg Mice			3xTg-AD Mice		
	6 Months	12 Months	16 Months	6 Months	12 Months	16 Months
**Quadriceps**						
**1. Nuclei**						
Localization	Peripheral nuclei	Peripheral nuclei	Peripheral nuclei	Peripheral nuclei	Peripheral nuclei	Peripheral nuclei
Number	++++	+++	+++	+++	++	+++
**2. Fiber**						
Distribution	Homogeneous	Homogeneous	Homogeneous	Homogeneous	Homogeneous	Homogeneous
**3. Adipose tissue**						
Localization	Intramuscular	Intramuscular	Intramuscular	Intramuscular	Intramuscular	Peripheral
Number	+	+	++	++	+	+
**Triceps surae**						
**1. Nuclei**						
Localization	Peripheral nuclei	Peripheral nuclei	Peripheral nuclei	Peripheral nuclei	Peripheral nuclei	Peripheral nuclei
Number	++++	+++	+++	+++	++	+++
**2. Fiber**						
Distribution	Homogeneous	Homogeneous	Heterogeneous	Homogeneous	Homogeneous	Heterogeneous
**3. Adipose tissue**		-				-
Localization	Intramuscular		Peripheral	Intramuscular	Intramuscular	
Number	+		++++	+	++++	
Qualifier: 75–100% = ++++. 50–75% = +++. 25–50% = ++. 0–25% = +. 0% = -.						

Table 2. Morphological features of muscle tissue in 3xTg-AD mice: localization and number of nuclei, fiber distribution, and a number and localization adipose cells. Qualitative quantifier of intensity: (-) equal to 0%, (+) less to 25%, (++) less to 50%, (+++) less to 75%, and (++++) less or 100%.

**Table 3:** Survival, kyphosis, and frailty phenotype.

Conditions	NTg Mice			3xTg-AD Mice			Statistics
	6 Months	12 Months	16 Months	6 Months	12 Months	16 Months	
**1. Survival** (mean + SEM days) (Mortality ratio)	329 + 25.26 3/15 (20%)	337 + 29.09 3/9 (33.3%)	350 + 15.60 20/40 (50%)	208 + 1.26 0/15 (0%)	395 + 9.63 1/16 (6.2%)	481 + 25.31 5/24 (20.8%)	**S ^&&^**
**2. Kyphosis** (animals, %)	-	3/6 (50%)	5/9 (56%)	1/6 (17%)	3/7 (43%)	4/11 (36%)	A **
Postural	-	-	-	1/6 (17%)	-	1/11 (9%)	**n.s.**
Structural	-	3/6 (50%)	5/9 (56%)	-	3/7 (43%)	3/11 (27%)	A*
**3. Physical conditions** (animals, %)							
Body weight	30 g.	30 g.	30 g.	28 g.	33 g.	34g.	A *, a ^#^
Alopecia	2/6 (33%)	4/6 (67%)	5/9 (56%)	1/6 (17%)	4/7 (57%)	4/11 (36%)	**n.s.**
Body position	-	-	-	-	-	5/11 (45%)	**a ^#^**
Palpebral closure	-	-	-	-	-	4/11 (36%)	**a ^#^**
Piloerection	-	1/6 (17%)	2/9 (22%)	-	-	6/11 (55%)	A *
Tail position	-	-	-	-	-	4/11 (36%)	**a ^#^**
Tremor	-	1/6 (17%)	-	-	-	9/11 (82%)	A **, G *
Kaplan–Meier, Log Rank: S ^&&^ *p* < 0.01. X_2_, A: age, ** *p* < 0.01 * *p* < 0.05, G: genotype, * *p* < 0.05. n.s. *p* > 0.05. ^#^ *p* < 0.05.							

Table 3. Prevalence of physical conditions in male 3xTg-AD mice corresponding to the frailty phenotype. The progression of AD disease is contrasted with normal aging and the survival of the experimental lots included in the research.

## Data Availability

Not applicable.
